# The cytochrome *bd*-I respiratory oxidase augments survival of multidrug-resistant *Escherichia coli* during infection

**DOI:** 10.1038/srep35285

**Published:** 2016-10-21

**Authors:** Mark Shepherd, Maud E. S. Achard, Adi Idris, Makrina Totsika, Minh-Duy Phan, Kate M. Peters, Sohinee Sarkar, Cláudia A. Ribeiro, Louise V. Holyoake, Dimitrios Ladakis, Glen C. Ulett, Matthew J. Sweet, Robert K. Poole, Alastair G. McEwan, Mark A. Schembri

**Affiliations:** 1 School of Biosciences, University of Kent, Canterbury, CT2 7NJ, United Kingdom; 2 School of Chemistry and Molecular Biosciences, The University of Queensland, Brisbane, Queensland 4072, Australia; 3 Australian Infectious Disease Research Centre, The University of Queensland, Brisbane, Queensland 4072, Australia; 4 School of Medical Science, and Menzies Health Institute Queensland, Griffith University, Gold Coast, Queensland, 4222, Australia; 5 Institute for Molecular Bioscience (IMB) and IMB Centre for Inflammation and Disease Research, University of Queensland, Brisbane, Queensland 4072, Australia; 6 Department of Molecular Biology and Biotechnology, The University of Sheffield, Firth Court, Western Bank, Sheffield, S10 2TN, United Kingdom

## Abstract

Nitric oxide (NO) is a toxic free radical produced by neutrophils and macrophages in response to infection. Uropathogenic *Escherichia coli* (UPEC) induces a variety of defence mechanisms in response to NO, including direct NO detoxification (Hmp, NorVW, NrfA), iron-sulphur cluster repair (YtfE), and the expression of the NO-tolerant cytochrome *bd*-I respiratory oxidase (CydAB). The current study quantifies the relative contribution of these systems to UPEC growth and survival during infection. Loss of the flavohemoglobin Hmp and cytochrome *bd*-I elicit the greatest sensitivity to NO-mediated growth inhibition, whereas all but the periplasmic nitrite reductase NrfA provide protection against neutrophil killing and promote survival within activated macrophages. Intriguingly, the cytochrome *bd*-I respiratory oxidase was the only system that augmented UPEC survival in a mouse model after 2 days, suggesting that maintaining aerobic respiration under conditions of nitrosative stress is a key factor for host colonisation. These findings suggest that while UPEC have acquired a host of specialized mechanisms to evade nitrosative stresses, the cytochrome *bd*-I respiratory oxidase is the main contributor to NO tolerance and host colonisation under microaerobic conditions. This respiratory complex is therefore of major importance for the accumulation of high bacterial loads during infection of the urinary tract.

Urinary tract infections (UTIs) are among the most common infectious diseases of humans and are the most common nosocomial infection in the developed world. They cause significant morbidity and mortality, with ~150 million cases globally per year^1^. Uropathogenic *Escherichia coli* (UPEC) cause the majority (~80%) of UTI in humans, including cystitis (bladder infection), pyelonephritis (kidney infection) and, in severe cases, urosepsis^1^. UPEC infection of the bladder results in the rapid influx of neutrophils and macrophages^2^, the activation of resident tissue macrophages and dendritic cells, and the release of proinflammatory cytokines^3^. Additionally, during infection UPEC encounters nitric oxide (NO), a membrane-soluble free radical that mediates its bactericidal effects via reaction with iron-sulphur clusters^4^, haem proteins^5^ and protein thiol groups^6^. A major source of NO is from host NO synthases, especially the inducible NO synthase (iNOS), which is activated in macrophages, for example, by bacterial lipopolysaccharide (LPS)^7^ and certain inflammatory cytokines, particularly interferon-gamma (IFN-γ)^8^. Other sources of NO include bacterial reduction of dietary nitrate, and the reactions of salivary nitrite with stomach acid to generate NO. There is no evidence for NOS activity in *E. coli*, although several bacteria including *Staphylococcus aureus*^9^, *Bacillus subtilis*^10^, *Helicobacter pylori*^11^ and *Streptomyces* species^12^ possess genes encoding NOS-like proteins.

Gram-negative bacteria can respond to NO-stress in a variety of ways, including detoxification via the flavohemoglobin Hmp^13^,^14^, the flavorubredoxin NorVW^15^, the nitrite reductase NrfA^16^, and the recently characterised NO reductase system Hcp/Hcr^17^. In addition, *E. coli* also utilises the diiron protein YtfE to repair iron-sulphur clusters damaged by nitrosative stress^18^, and possesses the NO-inducible cytochrome *bd*-I respiratory oxidase that confers resistance to NO^19^,^20^. Finally, efflux of glutathione and cysteine by the ABC transporter CydDC has also been shown to provide tolerance to nitric oxide^21^ (presumably via reaction of NO with these thiols), although the majority of the NO-tolerance effects result from the requirement of CydDC for the assembly of the NO-tolerant cytochrome *bd*-I terminal oxidase. The cytochrome *bd*-I complex, encoded by the *cydABX* operon, is expressed maximally in microaerobic environments under the dual control of the transcription factors ArcA and FNR^22^,^23^, and is up-regulated in response to NO^20^. Rather than catalyzing the decomposition of NO, cytochrome *bd*-I is an NO-tolerant terminal oxidase of the respiratory chain that permits aerobic respiration in the presence of NO and low oxygen^19^. Crucially, oxygen tension heavily influences the expression and efficacy of the above systems to provide protection against nitrosative stress^16^, and the relative importance of the mechanisms used to respond to NO stress for growth and survival of UPEC during microaerobic growth in the bladder remain to be fully elucidated. A recent transcriptomic study demonstrates that the cytochrome *bd*-I complex is highly-expressed in UPEC isolated from patients with UTI^24^, confirming that microaerobic conditions are encountered during infection, so the contribution of this system to NO tolerance is of particular interest to UPEC survival. In this study, we mutated the *hmp*, *cydAB*, *norVW*, *nrfA* and *ytfE* genes from a multidrug-resistant UPEC strain (EC958)^25^ from the recently emerged and globally disseminated ST131 lineage^26^,^27^. A *hcp/hcr* mutant was not constructed as this system is expressed only under anaerobic conditions, whereas the current study utilised microaerobic conditions to simulate conditions in the bladder. Mutant strains were assessed for growth inhibition in response to NO, for survival following neutrophil exposure, and for survival within activated macrophages. In addition, a mouse UTI model was used to assess the relative ability of the mutant strains to colonise the bladder. Our data indicate that loss of the cytochrome *bd*-I complex has the greatest impact upon the ability of UPEC to resist the challenges encountered during infection of the urinary tract.

## Results

### *E. coli* K-12 and clinical isolates exhibit similar sensitivity to an NO-releaser

To assess the sensitivity of UPEC to nitrosative stress, well diffusion assays were conducted with the NO releaser *S*-nitrosoglutathione (GSNO) using well-characterised UPEC strains associated with symptomatic (CFT073 and EC958) or asymptomatic (83972) infection, and the reference K-12 commensal strain MG1655. To simulate conditions during bladder infection, this experiment was conducted under a microaerobic atmosphere as microaerobically-expressed loci such as *cydAB* have previously been shown to be up-regulated during bladder infection^24^. The three UPEC strains exhibited a similar level of sensitivity to nitric oxide (EC958 = 12.7 ± 0.2 mm; CFT073 = 12.2 ± 0.2 mm; 83972 = 11.3 ± 0.2 mm) comparable to the sensitivity of MG1655 (10.3 ± 0.2 mm). Thus, toxicity to nitric oxide is conserved among the UPEC strains tested.

### Cytochrome *bd*-I and Hmp facilitate growth of EC958 in the presence of NO

**Figure 1:**
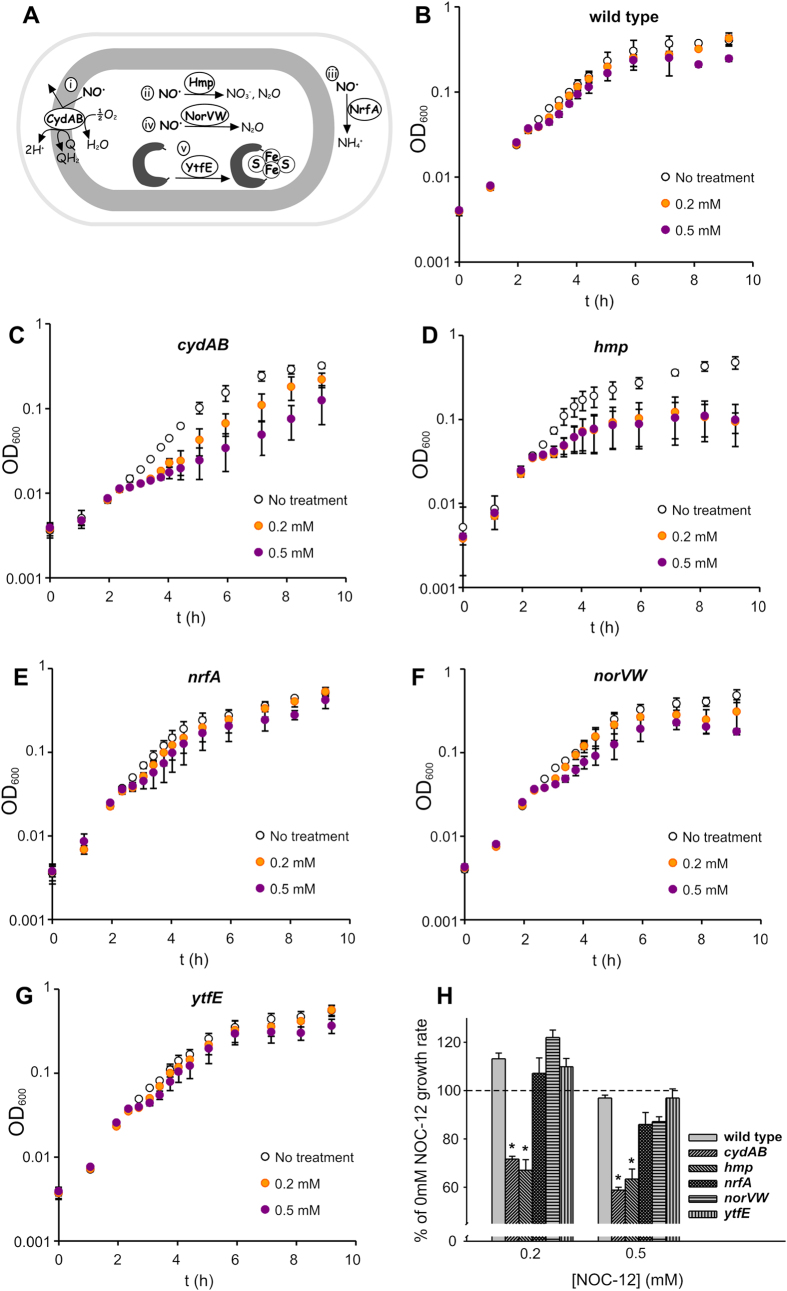
Loss of *cydAB* and *hmp* impairs growth in the presence of NO. (**A**) The NO-resistance mechanisms of *E. coli*: i) An NO-tolerant respiratory oxidase^19^ is induced (known as CydAB or cytochrome *bd*-I), which facilitates aerobic respiration under microaerobic conditions; ii) NO is converted to nitrate or nitrous oxide by the Flavohemoglobin Hmp under aerobic^55^ or anaerobic^56^ conditions, respectively; iii) NO is reduced by the periplasmic cytochrome *c* Nitrite Reductase NrfA^57^; iv) NO is converted to nitrous oxide via the Flavorubredoxin/Flavorubredoxin Reductase system NorVW^15^; v) The diiron protein YtfE repairs iron-sulphur clusters damaged by nitrosative stress^18^. (**B–G**) Cultures were grown under microaerobic conditions, and growth rates were measured following the addition of NOC-12 (0.2 mM and 0.5 mM). Error bars represent SD values. **(H)** Data from panels B-G are plotted as % growth rate compared to identical cultures grown in the absence of NOC-12. Error bars represent SEM. All data points are mean values calculated from five repeats. Asterisks indicate that rates measured in the presence of NOC-12 are significantly different from those measured in the absence of NOC-12 (Student’s *t*-test, *P* < 0.0001).

**Figure 2 f2:**
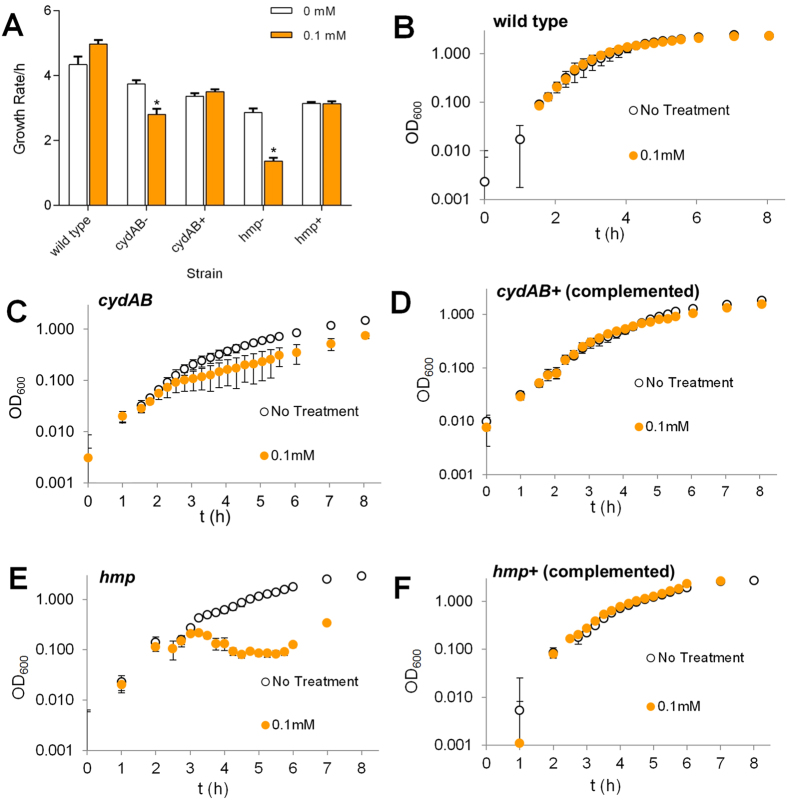
Complementation of NO-sensitive growth defects of *cydAB* and *hmp* mutants. *E. coli* EC958 knockout mutants of *cydAB* and *hmp* (EC958) were transformed with pSU2718 expression vectors (Table S2) containing the *hmp* and *cydABX* loci. Cultures (200 μl) were grown in M9 medium supplemented with casamino acids (0.1%) and glucose (4 g/L) in 96 well plates at 100 rpm in a Spectrostar Nano microplate reader (BMG Labtech). Growth rates were measured following the addition of NOC-12 (0 and 0.1 mM). The complemented *hmp* strain (*hmp*+) was grown in the presence of 1 mM IPTG, whereas the complemented *cydAB* strain (*cydAB*+) relied upon the basal expression of the *lac* promoter for expression of low levels of the *cydABX* operon (induction of this plasmid with IPTG was toxic to the cells). Panel A depicts the growth rates following NOC-12 additions, and panels B–F show the raw growth data used to calculate the growth rates in panel A. Mean data points and *P*-values are calculated from at least four independent replicates. Asterisks indicate that rates measured in the presence of NOC-12 are significantly different from those measured in the absence of NOC-12 (Student’s *t*-test, *P* < 0.05).

Gram-negative bacteria can respond to NO-stress in a variety of ways (Fig. 1A). To investigate which systems are important for tolerance to nitrosative stress in UPEC, genes encoding cytochrome *bd*-I (*cydAB*), Hmp, NorVW, NrfA and YtfE were deleted in EC958. Growth of wild type EC958, and *cydAB, hmp, nrfA, norVW* and *ytfE* mutants was monitored following addition of the NO-releaser NOC-12 (Fig. 1B–G). NOC-12 was preferred over GSNO in these experiments due to its NO-specific properties and amenability for use in small volume liquid growth experiments. Based on the NOC-12 growth data, mutation of *cydAB* and *hmp* conferred the greatest sensitivity to NO in the presence of 0.2 mM and 0.5 mM NOC-12 (Fig. 1H). This suggests that respiratory insensitivity to NO via cytochrome *bd*-I and NO-detoxification via the flavohemoglobin Hmp provide the greatest contribution to growth under these conditions. These growth defects were confirmed to result from loss of *cydAB* and *hmp* via complementation of the NO-sensitive phenotypes with plasmids containing the *hmp* and *cydABX* genes, respectively (Fig. 2). Cytochrome *bd*-I assembly was also monitored using whole cell CO difference spectroscopy. Using this approach, restoration of cytochrome *bd*-I assembly in the complemented *cydAB* mutant was verified based on its spectral features (Figure S1, Supplementary Information).

### NO resistance mechanisms of EC958 enhance survival in the presence of neutrophils and promote survival in macrophages

**Figure 3:**
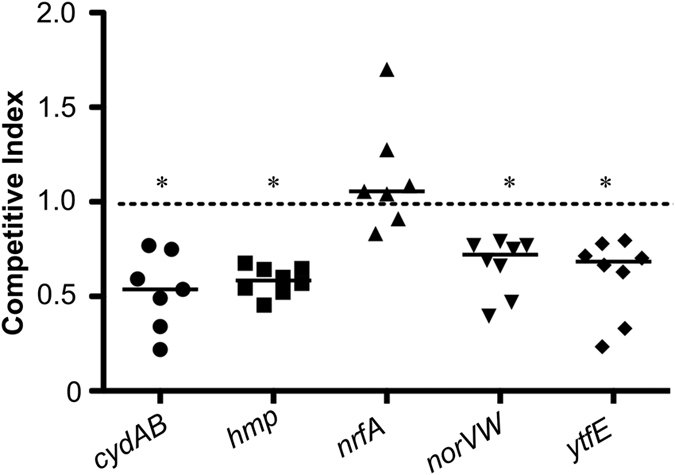
Loss of *cydAB*, *hmp*, *norVW*, and *ytfE* enhances killing by primary human neutrophils. Mixed infection data are plotted as a ratio of mutant:WT colonies. The horizontal bars represent mean values. These data are representative of three experiments using primary human neutrophils from different blood donors. Asterisks indicate mutant CFU data that are significantly different from WT (Student’s *t*-test, *P* < 0.05).

**Figure 4:**
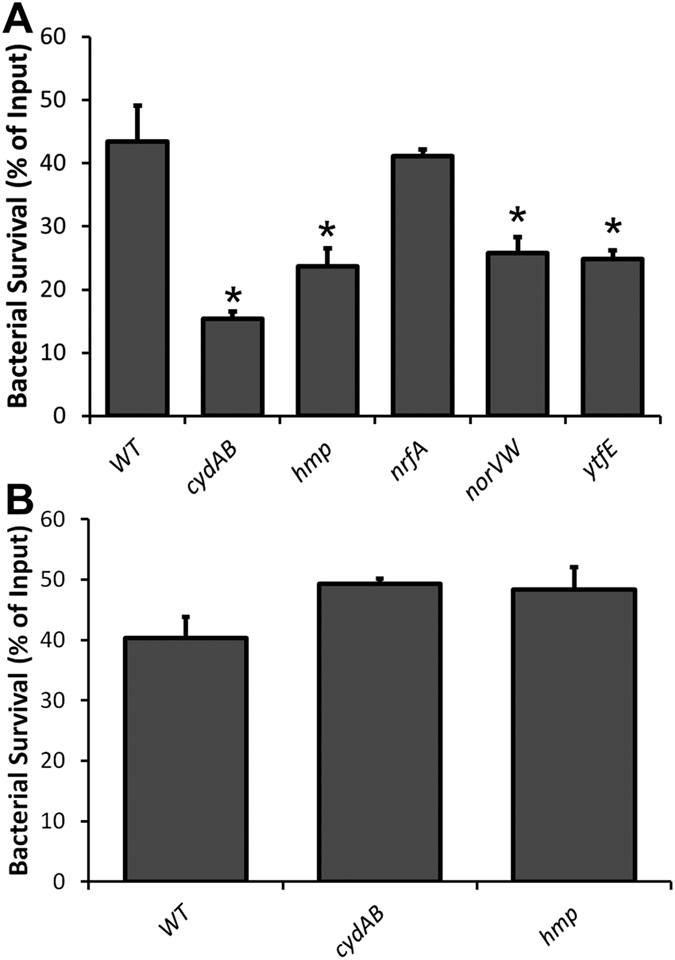
Loss of *cydAB*, *hmp*, *norVW*, and *ytfE* diminishes survival within primed macrophages. (**A**) Survival data using primed macrophages after 2 h are expressed as a % of intracellular bacteria at 20 min post-infection. (**B**) Control data with unprimed macrophages (experiments performed as in panel A) showing that loss of *cydAB* or *hmp* does not diminish survival. Data are representative of 3 independent infections, and error bars represent SEM. Asterisks indicate data that are significantly different from WT (Student’s *t*-test, *P* < 0.05).

Bacterial infection has previously been shown to induce nitric oxide synthase activity in human neutrophils^28^. The EC958 wild type, *cydAB, hmp, norVW, nrfA* and *ytfE* strains described above were tested for their ability to survive exposure to primary human neutrophils. In these experiments *cydAB*, *hmp*, *norVW* and *ytfE* mutants displayed increased sensitivity to neutrophil killing compared to wild type EC958, whereas loss of *nrfA* did not affect survival (Fig. 3). Similarly, infection of murine macrophages (lipopolysaccharide (LPS) and IFN-γ-activated to produce nitric oxide^29^) demonstrated that mutation of *cydAB*, *hmp*, *norVW* and *ytfE* led to a significant reduction in bacterial loads at 2 h post-infection, whereas loss of *nrfA* had no discernible effect (Fig. 4A). There was no decrease in survival of the *cydAB* and *hmp* mutant strains (compared to wild type) in the presence of non-primed macrophages (Fig. 4B), suggesting that NO is a major contributor to the killing of these mutant strains by macrophages. Furthermore, to confirm that primed macrophages were indeed producing NO, nitrite assays were undertaken (nitrite is a by-product of NO generation): nitrite could not be detected in non-primed cells whereas addition of both LPS and IFN-γ resulted in a nitrite concentration of 25 ± 0.8 μM. It is worth noting that this is a measure of accumulated nitrite pre-infection following 16 h of exposure to LPS/IFN-γ, and is not a measure of *in vivo* NO concentrations.

### Cytochrome *bd*-I contributes to EC958 survival in the mouse bladder

**Figure 5:**
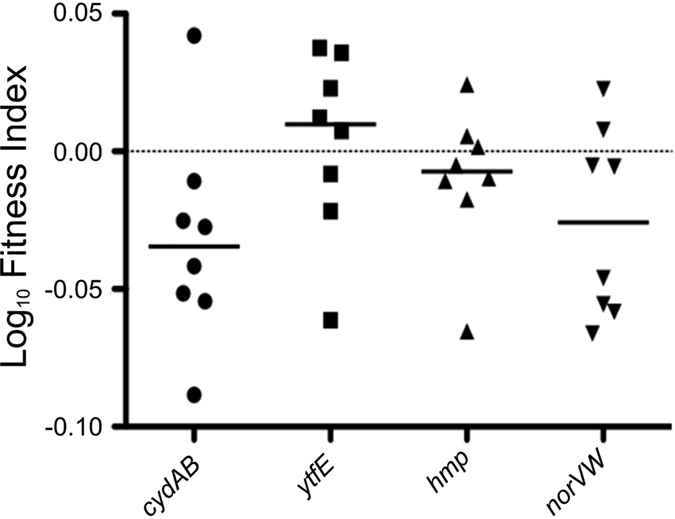
Deletion of *cydAB* impairs survival in a mouse UTI model. A minimum of eight C57BL/6 mice were transurethrally inoculated with ~5 × 10^8^ CFU of each strain. After 48 h, bladder homogenate samples were plated on MacConkey agar in triplicate for determination of bacterial loads. After 2 days, homogenated bladder samples were processed for bacterial loads by viable CFU counts performed in triplicate. Data are Log of the fitness index, which is defined as: Fitness index = (ratio of mutant:wild type after 2 days)/(initial mutant:wild type ratio). A minimum of 8 mice were included in each strain group. Equality of group medians was tested using the Wilcoxon matched pairs signed rank test.

To assess the contribution of defence mechanisms against NO to EC958 virulence, we tested the ability of the EC958 wild type, *cydAB, hmp, norVW* and *ytfE* mutants to survive in the mouse urinary tract using a competitive infection assay. We employed an EC958*lac* strain as the wild type to enable differentiation of both strains on MacConkey lactose medium; EC958*lac* had an identical growth rate in LB broth to wild type EC958^30^, produced type 1 fimbriae in similar levels and colonised the mouse bladder in equivalent numbers in a mixed competitive infection^31^. Female C57BL/6 mice were infected with 1:1 ratio of EC958*lac* and each mutant, respectively, and colonisation was assessed at 2-days post-infection. In these experiments, only the *cydAB* mutant displayed an attenuated colonisation phenotype (Fig. 5). EC958*cydAB* was significantly outcompeted by EC958*lac* in the bladder of infected mice (*P *= 0.0156, Wilcoxon matched pairs), whereas no significant competitive difference was observed in bacterial counts from the urine and kidneys of these mice (Figure S2, Supplementary Information).

## Discussion

It is well-known that UPEC encounter NO during infection^28^,^32^,^33^, so it was of interest to ascertain whether clinical isolates are more resistant to nitrosative stress compared to a well-characterised K-12 strain. The data presented herein clearly show that a range of UPEC isolates do not exhibit elevated resistance to the NO-releaser GSNO, which suggests that the molecular mechanisms used by UPEC to respond to nitrosative stress may be conserved. To our knowledge, all complete genome sequenced UPEC strains contain the core set of NO resistance genes that are also found in K-12 strains (i.e., *cydAB*, *hmp*, *nrfA*, *norVW*, *ytfE*). An extensive literature exists on the NO detoxification mechanisms of *E. coli* and is dominated by articles on the flavohemoglobin Hmp, reflecting the detoxification of NO to nitrate as an important process during infection. Indeed, the UPEC strain J96 has previously been shown to out-compete an isogenic *hmp* mutant in bladder and kidney colonisation in the mouse UTI model^14^, and pharmacological modulation of Hmp activity has recently been shown to elevate NO sensitivity in multidrug-resistant UPEC strains^34^. It is therefore reasonable to assume that Hmp provides considerable protection against nitrosative stress *in vivo*. However, the relative importance of other NO-tolerance mechanisms, such as iron-sulphur cluster repair or expression of an NO-tolerant respiratory oxidase, have never been assessed alongside Hmp for their contribution to *in vivo* survival. The current work provides novel insights into the relative importance of a range of mechanisms involved in NO-tolerance of UPEC in the context of the globally dominant multidrug-resistant ST131 clone.

The growth of knockout strains in the presence of the NO-releaser NOC-12 (Figs 1, [Fig f2] and 2) suggests that Hmp and cytochrome *bd*-I enhance the growth of UPEC under conditions of nitrosative stress and low oxygen, a similar environment to the bladder during UPEC infection^24^,^35^. These data display the growth rates following the addition of NOC-12, where diminished growth is likely to reflect a combination of bacteriostatic and bactericidal effects. Neutrophil killing assays (Fig. 3) indicate that all the systems under study, with the exception of NrfA, contribute to resistance against the nitrosative and oxidative burst produced by neutrophils. Given that loss of *nrfA* alone elicits marginal sensitivity to NO in *Salmonella*^16^ and that NrfA is only expressed under anaerobic conditions in the presence of nitrite/nitrate^36^, the behaviour of the *nrfA* mutant under microaerobic conditions used in the current study is unsurprising. In contrast, *ytfE* is up-regulated by nitrosative stress^20^,^37^ and has been shown to repair iron-sulphur clusters damaged by nitrosative and oxidative stresses^18^; thus our data are consistent with a role for this diiron protein in resistance against neutrophil killing. The *norVW*, *hmp* and *cydAB* genes all contributed to resistance against neutrophil killing, consistent with their previously characterised roles in nitric oxide tolerance.

Internalisation of the mutant strains by primed macrophages (Fig. 4A) reveals a similar pattern of survival to the neutrophil killing assays, mirroring the same hierarchical contribution towards survival. In support of a model in which nitrosative stress is likely responsible for diminished bacterial loads, bacterial survival was dramatically enhanced for the *cydAB* and *hmp* strains in the absence of LPS/IFN-γ stimulation (Fig. 4B), suggesting that iNOS activity contributes significantly to the killing of these mutants. The current data are consistent with the previous observation that Hmp enhances survival following internalisation by human macrophages^38^, and builds upon this work by adding cytochrome *bd*-I, NorVW, and YtfE to the list of systems that can provide similar protection. Together, the neutrophil and macrophage data suggest that cytochrome *bd*-I may offer the greatest resistance to sustained nitrosative stress, a conclusion that is supported by the mouse infection data (Fig. 5), where only the *cydAB* mutant was shown to exhibit diminished colonisation of the bladder. The absence of a phenotype for the *hmp* strain in the current mouse infection studies (Fig. 5) is a little surprising, as the *hmp* gene has previously been shown to confer a modest fitness advantage during colonisation of the urinary tract of C3H/HeN mice by a pyelonephritis-causing J96 *E. coli* strain (24 h post-infection)^14^. The absence of impaired bladder colonisation for the *hmp* strain in the current study may reflect differences in stress tolerance between the J96 strain previously used^14^,^38^,^39^ and the EC958 multidrug-resistant strain used herein^25^. In addition, differences in the mouse strains used (C3H/HeN in previous work^14^ and C57BL/6 mice in the current study) and timepoints for bacterial quantitation (24 h and 48 h post-infection for previous^14^ and current studies, respectively) may also be a contributory factor. Indeed, the infections had “generally cleared” by 48 h post-infection in the previous study^14^, potentially reflecting a more aggressive host response and/or a less resilient bacterial strain. If the bacterial strain is subjected to greater levels of nitrosative stress then loss of *hmp* is likely to have a more profound impact upon survival, which could explain differences in behaviour of *hmp* strains in the two studies. Nevertheless, the current data demonstrate that deletion of *cydAB* has a significant effect on bladder colonisation.

The recent observation that host-derived NO promotes UPEC uptake into bladder cells^40^ highlights the importance for NO tolerance as an important trait for UPEC during bladder colonisation, and the current data suggest that cytochrome *bd*-I facilitates this process. The transcription of the *cydAB* genes is also elevated in UPEC isolated from patients with UTI^24^, with mRNA transcript levels higher than those for *hmp*. Hence, cytochrome *bd*-I is likely to play a significant role as a dominant terminal electron acceptor of UPEC during colonisation of the urinary tract, enabling aerobic respiration to occur in the presence of NO levels that can inhibit other respiratory oxidases^19^. Clearly, the NO-tolerance mechanisms analysed in the current study may work in tandem to provide a concerted response to nitrosative stress over a range of environmental conditions. However, the current work suggests that the cytochrome *bd*-I terminal oxidase provides the greatest contribution to NO tolerance during growth in the bladder.

NO is also known to elevate biofilm dispersal in some Gram-negatives, and this process has been well-characterised in *Pseudomonas aeruginosa*^41^ and demonstrated more recently in *Salmonella enterica* and *E. coli*^42^. The ability to disperse biofilms and cell aggregates has clear clinical benefits, and recent combination approaches using NO and ciprofloxacin have proved an effective strategy to clear *P. aeruginosa* and *E. coli* O157 infections^43^. The current study provides insights into the relative importance of bacterial mechanisms of NO tolerance that are essential for the development of NO as a future antimicrobial therapy.

## Methods

### Ethical approval

This study was carried out in strict accordance with the recommendations in the Animal Care and Protection Act (Queensland, 2002) and the Australian Code of Practice for the Care and Use of Animals for Scientific Purposes (7th edition, 2004). Approval for mouse infection studies was obtained from the University of Queensland Animal Ethics Committee (SCMB/471/09/NHMRC (NF)). Approval for the collection of human blood was obtained from the University of Queensland Medical Research Ethics Committee (2008001123). All individuals provided written informed consent. All experimental protocols involving humans were carried out in accordance with the relevant guidelines and regulations.

### Bacterial strains and growth conditions

*E. coli* EC958 is a fluoroquinolone-resistant ST131 strain originally isolated from the urine of a patient with UTI in the United Kingdom in 2005^25^,^44^, and EC958*lac* is a previously described tagged derivative strain used to enable differential selection on MacConkey lactose agar^30^. CFT073 is a blood culture isolate from a patient with pyelonephritis (CFT073)^45^, 83972 is a urine isolate from an individual with asymptomatic bacteruria^46^, and MG1655 is a K-12 commensal strain^47^. *E. coli* strains were routinely cultured at 37 °C on solid or in liquid Luria-Bertani (LB) media supplemented with appropriate antibiotics as required (20 μg/ml gentamycin, 30 μg/ml chloramphenicol).

### Construction of mutant strains and plasmids

Inactivation of the *cydAB, hmp, norVW, nrfA,* and *ytfE* genes in EC958 was performed as previously described^25^. The chloramphenicol resistance cassette was amplified from plasmid pKD3^25^ using primers containing 50 nucleotide flanking regions complementary to the beginning and end of the target loci. The knock-out PCR product was introduced by electroporation into EC958 harbouring a gentamycin-resistant plasmid carrying the *λ*-Red recombinase. Allelic exchange inactivation of the target genes was performed as previously described^48^ and the constructed mutations were confirmed by sequencing of the mutated sites. All primers, strains and plasmids are listed in Tables S1–S3 (Supplementary Information).

### Assessment of NO sensitivity

NO sensitivity was assessed on solid medium using a well diffusion assay with the NO-releaser GSNO. GSNO was prepared as previously described^49^ and quantified using the extinction coefficient ε_545_ = 15.9 M^−1^cm^−1^ 50. Strains were grown aerobically at 37 °C in M9 minimal medium (16 g/l Na_2_HPO_4_.2H_2_O, 3 g/l KH_2_PO_4_, 0.5 g/l NaCl, 1 g/l NH_4_Cl, 0.24 g/l MgSO_4_, 0.01 g/l CaCl_2_, and 4 g/l glucose) supplemented with 0.1% casaminoacids, from a 1% inoculum of an overnight aerobic culture in LB media. When mid-log phase was reached, cells were plated in M9 minimal agar medium using the pour plate technique. Six 6 mm diameter-wells were cut in the agar gel and 80 μl of a 105 mM GSNO solution was added to each. Plates were incubated at 37 °C in microaerobic (2% oxygen) conditions in a Ruskinn InvivO_2_ hypoxia workstation. Average zones of inhibition (± SEM) were calculated from six repeats.

NO-sensitivity was assessed in liquid medium using growth curves in the presence of the slow NO-releaser NOC-12 (t_1/2_ = 100 min at 37 °C, pH 7.4) as previously described^19^. Briefly, cultures (200 μL) were grown in 96-well plates (37 °C, 160 rpm) in M9 medium supplemented with casamino acids (0.1%) and glucose (2 g/L). NOC-12 was suspended in 50 mM sodium phosphate pH 8.0 immediately before use and added to the culture when A_600_ reached 0.004. Growth rates were calculated from subsequent readings over the next 1.5 h.

### Neutrophil killing assay

Cultures of EC958 and each of the five knockout strains (Table S3, Supplementary Information) were grown overnight in LB under conditions of low aeration, and primary neutrophils were freshly prepared from human peripheral blood the following day by discontinuous density sedimentation as previously described^51^. Competitive infections were performed with wild type EC958 and each of the five knockout strains. In each case, bacterial strains were mixed at a ratio of 1:1 and used at a final multiplicity of infection (MOI) of 10:1 (bacteria:neutrophil). Data are representative of three independent infections and the error bars represent SEM of the replicates in a single experiment performed with neutrophils. Survival was assessed at 30 min post-infection via plating with and without chloramphenicol to enumerate total CFU and mutant CFU, respectively. The significance of differences between the wild type strain and the mutant strains were assessed using the Student’s *t*-test.

### Intracellular macrophage survival assay

Single infection of pre-primed RAW264.7 cells was performed essentially as previously described^52^ at a multiplicity of infection (MOI) of 10 bacteria:1 macrophage. Cells were primed with 0.5 ng/ml of IFN-γ and 10 ng/ml LPS for 16 h prior to infection. After a 10 min infection period extracellular bacteria were eliminated via gentamycin (indicate concentration used) treatment for 10 min. Macrophages were washed and lysed with 0.01% Triton X-100 at 20 min or at 2 h post-infection. Intracellular bacterial loads (CFU/ml) were assessed via plating on LB agar. Survival data are presented as a percentage of CFU present at t = 2 h compared to CFU at 20 min. Data are representative of 3 independent infections and the error bars represent the range of error of the replicates in a single experiment.

### Bladder colonisation in a Mouse UTI model

All bacterial strains were enriched for the expression of type I fimbriae using static growth as previously described^25^. The C57BL/6 mouse model of UTI was used to assess *in vivo* survival as previously described^53^. Briefly, female C57BL/6 mice (8–10 weeks) were transurethrally inoculated^54^ with ~5 × 10^8^ CFU using a 1 ml tuberculin syringe attached to a sterile catheter. After 2 days, urine and homogenated bladder and kidney samples were processed for bacterial loads by viable CFU counts (performed in triplicate). The wild type strain had the *gfp* gene inserted into the *lac* operon (Table S3, Supplementary Information), so wild type could be discriminated from mutant by their appearance on MacConkey agar. Data are displayed as Log_10_ of the fitness index, which is defined as: Fitness index = (ratio of mutant:wild type after 2 days)/(initial mutant:wild type ratio). A minimum of 8 mice were included in each strain group. Equality of group medians was tested using the Wilcoxon matched pairs signed rank test.

## Additional Information

**How to cite this article**: Shepherd, M. *et al*. The cytochrome *bd*-I respiratory oxidase augments survival of multidrug-resistant *Escherichia coli* during infection. *Sci. Rep.* **6**, 35285; doi: 10.1038/srep35285 (2016).

## Supplementary Material

Supplementary Information
