# Supplementary material for: The cytochrome bd-I respiratory oxidase augments survival of multidrug-resistant Escherichia coli during infection
Source: Sci Rep. 2016 Oct 21;6:35285. doi: 10.1038/srep35285 (PMC5073308; doi:10.1038/srep35285)
Supplement: Supplementary Information [file srep35285-s1.pdf]

The cytochrome *bd*-I respiratory oxidase augments survival of multidrug-resistant *Escherichia coli* during infection

Mark Shepherd[1*], Maud E. S. Achard[2,3], Adi Idris[2,3†], Makrina Totsika[2,3#], Minh-Duy Phan[2,3], Kate M. Peters[2,3], Sohinee Sarkar[2,3#], Claudia A. Ribeiro[1], Louise V. Holyoake[1], Dimitrios Ladakis[1], Glen C. Ulett[4], Matthew J. Sweet[5], Robert K. Poole[6], Alastair G. McEwan[2,3], Mark A. Schembri[2,3*]

[1]School of Biosciences, University of Kent, Canterbury, CT2 7NJ, United Kingdom
[2]School of Chemistry and Molecular Biosciences, The University of Queensland, Brisbane, Queensland 4072, Australia
[3]Australian Infectious Disease Research Centre, The University of Queensland, Brisbane, Queensland 4072, Australia
[4]School of Medical Science, and Menzies Health Institute Queensland, Griffith University, Gold Coast, Queensland, 4222, Australia
[5]Institute for Molecular Bioscience (IMB) and IMB Centre for Inflammation and Disease Research, University of Queensland, Brisbane, Queensland 4072, Australia
[6]Department of Molecular Biology and Biotechnology, The University of Sheffield, Firth Court, Western Bank, Sheffield, S10 2TN, United Kingdom

*M.Shepherd@kent.ac.uk; m.schembri@uq.edu.au

#Current address: Institute of Health and Biomedical Innovation, and School of Biomedical Sciences, Queensland University of Technology, Kelvin Grove, Queensland 4059, Australia.
†Current address: PAPRSB Institute of Health Science, Universiti Brunei Darussalam, Brunei Darussalam, BE1410

Figure S1. Complementation of the *cydAB* mutant restores the *bd*-type spectroscopic signal
Wild type, *cydAB*, and *cydAB*+ (complemented mutant – see Tables S2 and S3 for strains and plasmids) strains were grown in 250 mL conical flasks in 100 mL LB medium overnight at 37°C and 150 rpm. Cells (from 30 mL of culture) were harvested and CO difference spectra were recorded as previously described (Kalnenieks, *et al*. FEMS Microbiol. Lett. 1998;168(1):91-97). The expected spectral features for cytochrome *bd*-I are troughs at 430 nm, 442 nm, and 622 nm, and a peak at 642 nm (Kita *et al*. J. Biol. Chem. 259:3375-3381 (1984)).

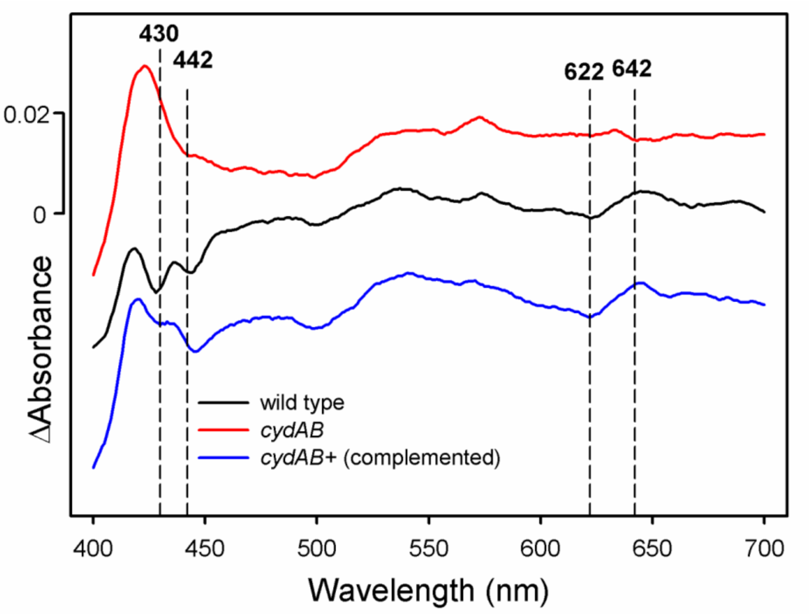

Figure S2. Loss of *cydAB*, *hmp*, *norVW* or *ytfE* does not decrease bacterial load in the urine or kidney
A minimum of eight C57BL/6 mice were transurethrally inoculated with ~5x10^8 CFU of each strain. After 48 h, bladder homogenate samples were plated on Maconkey agar in triplicate for determination of bacterial loads. After 2 days, urine (panel A) and homogenated kidney samples (panel B) were processed for bacterial loads by viable CFU counts performed in triplicate. Data are Log of the fitness index, which is defined as: Fitness index = (ratio of mutant:wild type after 2 days)/(initial mutant:wild type ratio). A minimum of 8 mice were included in each strain group. Equality of group medians was tested using the Wilcoxon matched pairs signed rank test.

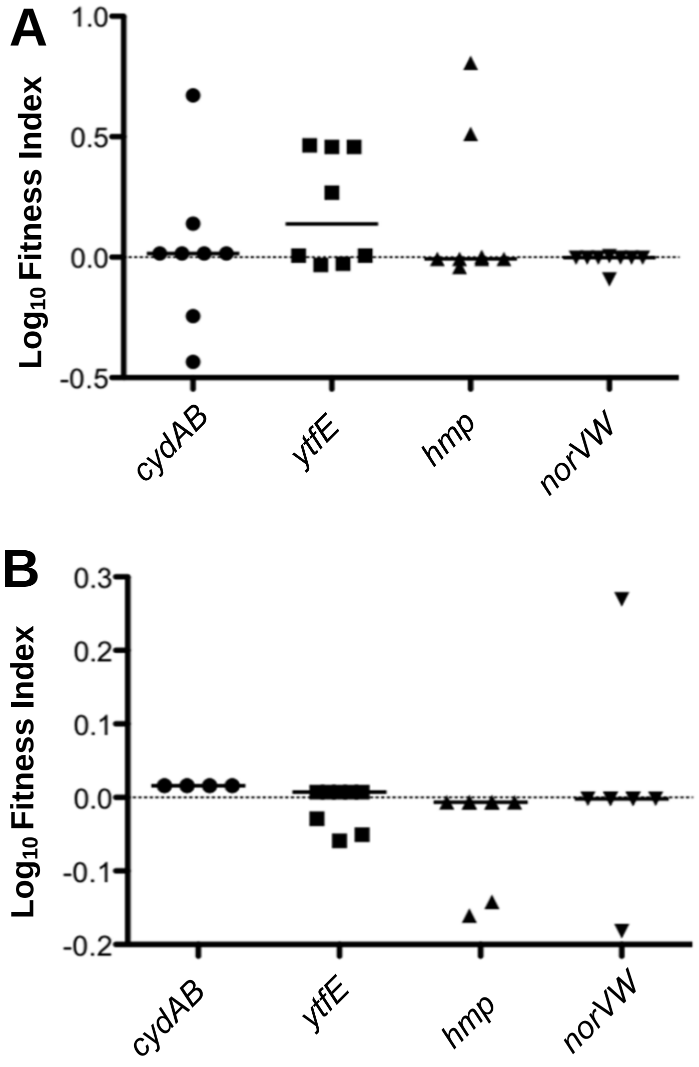

Table S1. Mutagenesis primers

Primer	Sequence (5´ – 3´)	Description	Reference or source
cydA CmF	**ATGATGTTAGATATAGTC GAACTGTCGCGCTTACAG TTTGCCTTGACCGC**GTGT AGGCTGGAGCTGCTTC	Forward mutagenesis primer comprising 50 bases complementary to the start of *cydA* followed by 20 bases complementary to the Cm cassette of pKD3.	This work
cydB CmR	**TTAGTACAGAGAGTGGGT GTTACGTTCAATATCTTC TTTGGTGATACGAC**CATA TGAATATCCTCCTTAG	Reverse mutagenesis primer comprising 50 bases complementary to the end of *cydB* followed by 20 bases complementary to the Cm cassette of pKD3.	This work
hmp CmF	**ATGCTTGACGCTCAAACC ATCGCTACAGTAAAAGCC ACCATCCCTTTACT**GTGTA GGCTGGAGCTGCTTC	Forward mutagenesis primer comprising 50 bases complementary to the start of *hmp* followed by 20 bases complementary to the Cm cassette of pKD3.	This work
hmp CmR	**TCACAGCACCTTATGCGG GCCAAAGCATTCGTAATG AATGTTTTCCTGTT**CATAT GAATATCCTCCTTAG	Reverse mutagenesis primer comprising 50 bases complementary to the end of *hmp* followed by 20 bases complementary to the Cm cassette of pKD3.	This work
nrfA CmF	**ATGACAAGGATAAAAATA AACGCACGCCGTATCTTC AGCTTATTGATTCC**GTGTA GGCTGGAGCTGCTTC	Forward mutagenesis primer comprising 50 bases complementary to the start of *nrfA* followed by 20 bases complementary to the Cm cassette of pKD3	This work
nrfA CmR	**TTATTGGCTTAACAGACC GTTTTTACGTGCCTGCTC TTCCCACTGCGGGA**CATA TGAATATCCTCCTTAG	Reverse mutagenesis primer comprising 50 bases complementary to the end of *nrfA* followed by 20 bases complementary to the Cm cassette of pKD3.	This work
norV CmF	**ATGTCTATTGTGGTGAAA AATAACATTCATTGGGTT GGTCAACGTGACTG**GTGT AGGCTGGAGCTGCTTC	Forward mutagenesis primer comprising 50 bases complementary to the start of *norV* followed by 20 bases complementary to the Cm cassette of pKD3.	This work
norW CmR	**GTTTTCAACAATCCAAAT GCCTCTTTCATCCGATCC TCACTGACCACAAA**CATA TGAATATCCTCCTTAG	Reverse mutagenesis primer comprising 50 bases complementary to the end of *norW* followed by 20 bases complementary to the Cm cassette of pKD3.	This work
ytfE CmF	**ATGGCTTATCGCGACCAA CCTTTAGGTGAACTGGCG CTCTCTATCCCTCG**GTGTA GGCTGGAGCTGCTTC	Forward mutagenesis primer comprising 50 bases complementary to the start of *ytfE* followed by 20 bases complementary to the Cm cassette of pKD3.	This work
ytfE CmR	**TTACTCACCCGCCAGCGC GCGTGGGAACAGTACATT GTTTTCCAGGCTGA**CATA TGAATATCCTCCTTAG	Reverse mutagenesis primer comprising 50 bases complementary to the end of *ytfE* followed by 20 bases complementary to the Cm cassette of pKD3.	This work

Table S2. Cloning primers and plasmids

Strain	Sequence (5´ – 3´)	Description	Reference or source
cyd F	ACTCTCGGATCC TTAAAGAGGAGAAAGGTACCGC ATGTTAGATATAGTCGAACTGTCGC	Forward cloning primer for *cydABX* and *ygbE*, contains BamHI site (underlined)	This work
cyd R	AGGGAGTCTAGAAATTTATGGGCC TGACTCGG	Reverse cloning primer for *cydABX* and *ygbE*, contains XbaI site (underlined)	This work
hmp F	CGCGCGGATCCATTTTTCACTTCAT CACATTCTTTCTGAAA	Forward cloning primer for *hmp*, contains BamHI site (underlined)	This work
hmp R	CGCGCTGCGCATGCCGGATGTGTCC ATCCGGCAACATCAAA	Reverse cloning primer for *hmp*, contains FspI site (underlined)	This work

Plasmid	Description	Reference or source
pSU2718-G	A gentamycin resistance cassette was cloned into the chloramphenicol resistance cassette of vector pSU2718. For use in complementation studies in multidrug-resistant strains.	This work
pSU2718G-*cydABX*	The *cydABX* operon (with ribosome binding site) and the downstream *ygbE* gene were cloned into the BamHI/XbaI sites of vector pSU2718-G.	This work
pSU2718-G-*hmp*	The *hmp* gene, including 120 bp of native promoter, was cloned into the BamHI/FspI sites of vector pSU2718-G.	This work

Table S3. *E. coli* strains

Strain	Description	Reference or source
EC958	O25:H4-ST131. Multidrug-resistant (referred to as wild type)	Lau *et al.* J. Antimicrob. Chemother. 62: 1241-1244 (2008).
cydAB	As EC958, except *cydAB*::ΩCm^{R}	This work
hmp	As EC958, except *hmp*::ΩCm^{R}	This work
norVW	As EC958, except *norVW*::ΩCm^{R}	This work
nrfA	As EC958, except *nrfA*::ΩCm^{R}	This work
ytfE	As EC958, except *ytfE*::ΩCm^{R}	This work
EC958*lac*	As EC958, except *lacZ*::Ω*gfp*	This work
CFT073	Pyelonephritis isolate	Mobley, H.L. *et al. Infect. Immun.* 58: 1281-1289 (1990).
83972	Asymptomatic bacteriuria isolate	Andersson, P. *et al. Infect. Immun.* 59: 2915-2921 (1991).
MG1655	Nonpathogenic K12 strain	Blattner, F.R. *et al. Science* **277**, 1453-1462 (1997).
